# Mapping causal circuit dynamics in stroke using simultaneous electroencephalography and transcranial magnetic stimulation

**DOI:** 10.1186/s12883-021-02319-0

**Published:** 2021-07-16

**Authors:** Camarin E. Rolle, Fiona M. Baumer, Joshua T. Jordan, Ketura Berry, Madelleine Garcia, Karen Monusko, Hersh Trivedi, Wei Wu, Russell Toll, Marion S. Buckwalter, Maarten Lansberg, Amit Etkin

**Affiliations:** 1 Department of Psychiatry and Behavioral Sciences, Stanford University School of Medicine, 401 Quarry Road, MC: 5797, Stanford, CA 94305-5797 USA; 2 Wu Tsai Neurosciences Institute, Stanford University, Stanford, CA USA; 3 Sierra-Pacific Mental Illness Research, Education, and Clinical Centers (MIRECC), Palo Alto Veterans Health Care Administration, Palo Alto, CA USA; 4 Department of Neurology and Neurological Sciences, Stanford University School of Medicine, Stanford, CA USA; 5 Department of Psychiatry, University of California At San Francisco, San Francisco, CA USA; 6 School of Medicine, University of California, San Francisco, San Francisco, CA USA; 7 Department of Neurosurgery, Stanford University School of Medicine, Stanford, CA USA

**Keywords:** Stroke, TMS-EEG, Connectivity, Motor cortex, Beta, wPLI

## Abstract

**Background:**

Motor impairment after stroke is due not only to direct tissue loss but also to disrupted connectivity within the motor network. Mixed results from studies attempting to enhance motor recovery with Transcranial Magnetic Stimulation (TMS) highlight the need for a better understanding of both connectivity after stroke and the impact of TMS on this connectivity. This study used TMS-EEG to map the causal information flow in the motor network of healthy adult subjects and define how stroke alters these circuits.

**Methods:**

Fourteen stroke patients and 12 controls received TMS to two sites (bilateral primary motor cortices) during two motor tasks (paretic/dominant hand movement vs. rest) while EEG measured the cortical response to TMS pulses. TMS-EEG based connectivity measurements were derived for each hemisphere and the change in connectivity (ΔC) between the two motor tasks was calculated. We analyzed if ΔC for each hemisphere differed between the stroke and control groups or across TMS sites, and whether ΔC correlated with arm function in stroke patients.

**Results:**

Right hand movement increased connectivity in the left compared to the right hemisphere in controls, while hand movement did not significantly change connectivity in either hemisphere in stroke. Stroke patients with the largest increase in *healthy hemisphere connectivity* during paretic hand movement had the best arm function.

**Conclusions:**

TMS-EEG measurements are sensitive to movement-induced changes in brain connectivity. These measurements may characterize clinically meaningful changes in circuit dynamics after stroke, thus providing specific targets for trials of TMS in post-stroke rehabilitation.

## Background

Stroke is a leading cause of death and long-term disability in the United States [1]. Motor impairments affect 85% of stroke patients initially and persist in 50% [2]. To develop effective therapies for these motor deficits, we need a better understanding of the neurophysiology of stroke recovery. Functional magnetic resonance imaging (fMRI) shows that immediately after stroke, movement of the paretic hand activates the bilateral motor cortices instead of activating primarily the contralateral motor cortex. Persistence of bilateral activation correlates with poor recovery [3, 4], but it is unclear if this is a pathologic pattern hindering recovery or a compensatory mechanism in patients with severe injury [5–7].

Several studies using fMRI [8–10] and transcranial magnetic stimulation (TMS) [11, 12] have examined the interactions between the two hemispheres after stroke. These methods are complementary: fMRI has high spatial specificity but relies on correlative data, whereas TMS is unique in its ability to directly test the downstream influence of the stimulated brain region [13]. Typically, hand movement is associated with an increase in contralateral motor cortex activation paired with inhibition of the ipsilateral motor cortex [14]. After stroke, the motor cortex ipsilateral to paretic hand movement inhibits activity of the injured, contralateral one [14]. Numerous neuromodulatory trials using repetitive TMS have attempted to reinstate a normal “inhibitory balance” between the hemispheres, with mixed results [15, 16]. Furthermore, the TMS-fMRI work of Bestmann et al. [5] suggests that in the most severely affected chronic stroke patients, the ipsilateral, healthy hemisphere may actually facilitate paretic hand movement (for review, see [17, 18]). These complex and patient-specific interactions highlight a need for better tools to measure bihemispheric dynamics after stroke and the influence of therapy on them.

TMS paired with electroencephalography (TMS-EEG) offers an opportunity to interrogate cortico-cortical interactions between the motor cortices after stroke with high temporal precision [19]. Single TMS pulses (spTMS) introduce a cortical current, the downstream effect of which can be mapped by EEG on the order of milliseconds [20–22]. Synchronization and desynchronization of the EEG oscillations evoked from TMS single pulses reflect excitability and anatomo-functional properties of the stimulated cortex [23, 24]. Therefore, if stimulation of one brain region alters synchronization of EEG activity within another (e.g. local connectivity), we can infer that the stimulated region causally influences the measured region [25]. Furthermore, TMS can be administered during various tasks to directly interrogate how a task (e.g. hand movement) changes connectivity between the stimulated and measured regions [26, 27].

In this study, we sought to characterize changes in brain activity induced by hand movement and to determine if these metrics relate to motor function after stroke. We specifically investigated this question using TMS-EEG to determine if TMS-evoked spectral metrics distinguish movement from rest, supplementing the existing state-dependent fMRI and TMS-fMRI characterization of brain activation following stroke [8–12]. Informed by the prior literature [8–12], we hypothesized that hand movement would selectively increase connectivity in the contralateral hemisphere compared to the ipsilateral hemisphere in controls and that this lateralization would be altered in stroke. Thus, we stimulated the bilateral primary motor cortices (M1) while subjects were engaged in a motor task and at rest. We measured each hemisphere’s response to TMS by calculating the debiased weighted-phase lag index (wPLI) – a robust measure of phase synchronization and connectivity that is minimally affected by volume conduction [28–30]. We specifically focused on wPLI estimates within the beta frequency band given its significance in motor function [31–34].

## Materials and methods

### Participants

Fourteen right-handed patients with a history of a single ischemic stroke (9 Female; 8 right & 6 left hemisphere strokes) were recruited from the Stanford Stroke Center (Table 1). Inclusion criteria included: (i) age > 18 years; (ii) history of ischemic stroke verified by MRI; (iii) only one lifetime stroke; (iv) right-handedness; and (v) persistent unilateral upper extremity motor deficit (regardless of severity of deficit). Exclusion criteria included: (i) contraindications to TMS (i.e. epilepsy); (ii) severe aphasia limiting ability to consent; (iii) hemorrhagic stroke; and (iv) multifocal infarcts. One participant was in the sub-acute phase of stroke recovery (2 months post-stroke) while the other 13 were in the chronic phase (> 6 months post-stroke) [35]. Demographic and clinical information was gathered from the subjects and their clinical chart. The laterality, location, and volume of the stroke lesions was determined by review of clinical imaging. Participants had variable degrees of motor weakness, so on the day of the TMS session, they underwent the Fugl-Meyer assessment to quantify the severity of their motor impairment [36]. Three participants refused the Fugl-Meyer assessment, and a score was imputed by an experienced occupational therapist (JW) based on records from the clinic visit proximate to study participation [37]. In addition, twelve right-handed age-matched control participants (6 Female) with no contraindications to TMS were recruited from the local community. The Stanford University Institutional Review Board approved the protocol and participants provided written informed consent. All methods were performed in accordance with the relevant guidelines and regulations.

**Table 1:** Characteristics of study sample

**Subject Characteristics**	**Stroke (*n* = 14)**	**Controls (*n* = 12)**
Demographics		
Age (yr, mean ± SD)	56 ± 16	54 ± 16
Age Range (yr)	33–85	28–74
Gender (%male)	64%	50%
Stroke characteristics		
Stroke Side (n, %)		
Left	6 (43%)	
Right	8 (57%)	
Stroke Location (n, %)		
Cortical & Subcortical	11 (79%)	
Subcortical Only	3 (21%)	
Time Since Stroke (yrs, mean ± SD)	3.4 ± 2.5	
Stroke Volume (ml, mean ± SD)	65 ± 65	
Paretic arm function		
Fugl-Meyer Score (mean ± SD)	32 ± 22	
Finger-Tapping Speed (Taps/min)		
Paretic Hand	6 ± 10	
Non-Paretic Hand	34 ± 15	

### Experimental set-up

#### Motor tasks

**Fig. 1:**
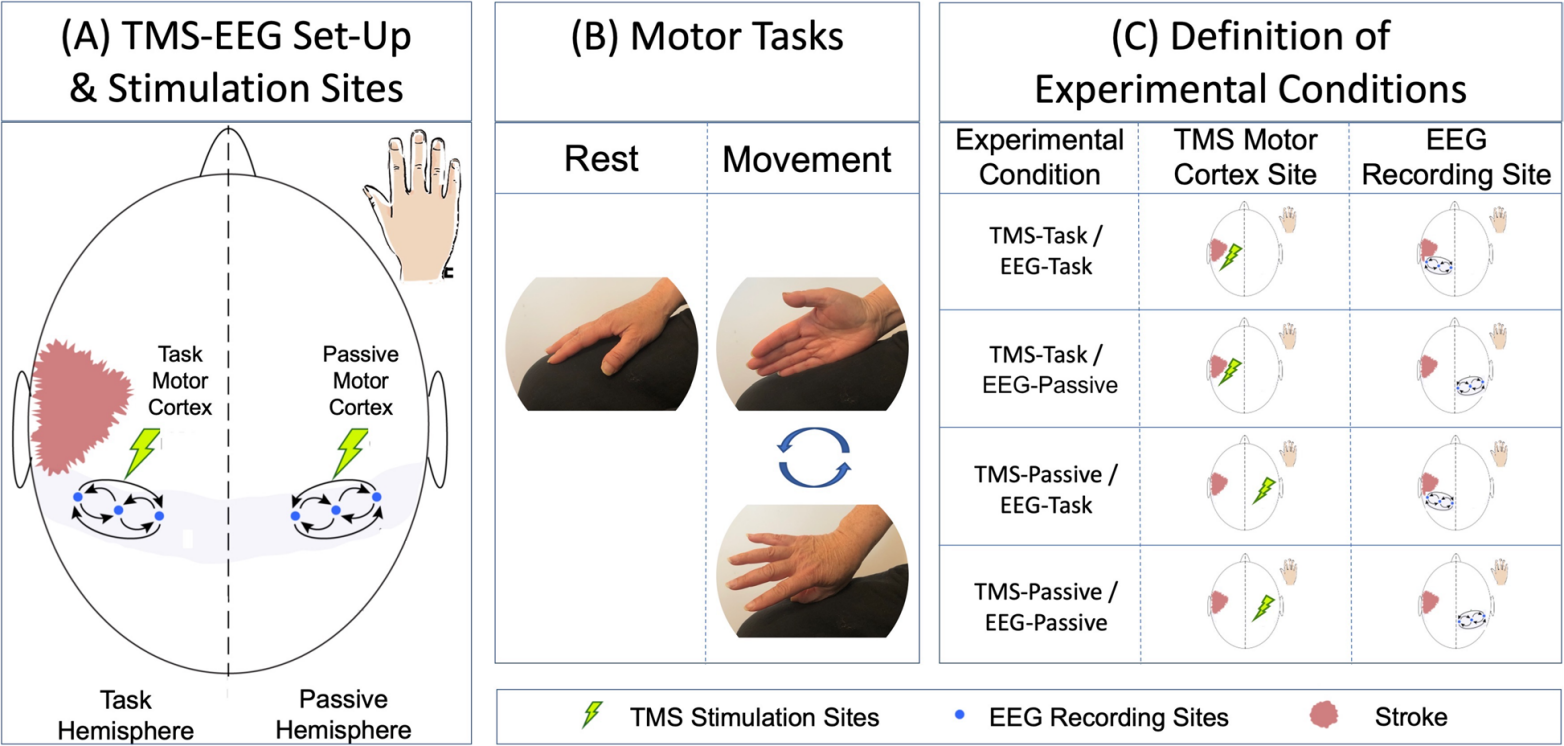
Experimental Set-up & Terminology. **A** Subjects received 60 stimuli at 120% resting motor threshold to each primary motor cortex (green lightning bolts) and EEG signal was measured from a 64-channel cap with special attention paid to electrodes overlying each motor cortex (blue dots). **B** TMS was applied during two motor tasks (rest & movement). Stroke subjects performed motor tasks with the paretic hand, while control subjects performed tasks with the dominant (right) hand. Connectivity was quantified within each hemisphere by computing synchronization of signal in electrodes overlying each motor cortex and the change in connectivity (ΔC) between the two motor tasks was calculated. **C** The hemisphere contralateral to the moving hand was termed the “Task Hemisphere” while the hemisphere ipsilateral to the moving hand was the “Passive Hemisphere.” There were thus 4 combinations of TMS-EEG recordings, termed Experimental Conditions: TMS-Task/EEG-Task, TMS-Task/EEG-Passive, TMS-Passive/EEG-Task, TMS-Passive/EEG-Passive

Patients sat in a reclining chair, facing a fixation cross displayed on a computer monitor at eye level to center attention and performed 2 motor tasks (Fig. 1). During the resting task, participants kept their hands still, palms down on their legs. During the hand-movement task, participants continuously rotated the hand at the wrist, alternating between tapping the leg with the thumb and fifth finger. Controls moved their dominant (right) hand and stroke patients were asked to move their paretic hand; stroke participants unable to rotate the hand (8/14 participants; 57.1%) were asked to visualize this movement, which has been shown to produce comparable cortical activations in the primary motor cortex [38].

#### TMS

TMS was administered using a Cool-B B65 butterfly coil and a MagPro X100 TMS stimulator (MagVenture, Denmark), and Visor2 LT 3D neuronavigation system (ANT Neuro, Netherlands). Individual anatomical MRIs (T1-weighted, 3 T, slice distance 1 mm, slice thickness 1 mm, sagittal orientation, acquisition matrix 256 × 256 acquired with a 3 T GE DISCOVERY MR750 scanner) were acquired and the bilateral motor cortex stimulation sites were defined by the anatomical atlas correspondence with the MNI template. All MRI scans used to guide TMS targeting were reviewed independently by two neurologists (FMB, ML) to confirm that the primary motor cortex targets were intact in all stroke subjects.

During TMS, the coil was placed tangentially to the scalp with the handle pointing backwards and laterally angled 45° from the sagittal plane. Stimulation parameters included biphasic TMS pulses, 280 µs pulse width, 1500 ms recharge delay, interleaved at a random interval of 3 s ± 300 ms. A thin foam pad was attached to the coil surface to decrease electrode movement and dampen coil vibration while white noise was used to mask the TMS click. Resting motor threshold (rMT), defined as the minimum stimulation intensity required to produce visible twitch of the hand muscles in 50% of trials [39], was determined for the primary motor cortex of the dominant hemisphere in controls and the lesioned hemisphere in stroke patients. We ensured using neuro-navigation that the cortical regions stimulated and those underlying the EEG electrodes were not directly injured by the stroke itself and all participants had an elicitable motor evoked potential. Two cortical targets (left and right primary motor cortex) were stimulated with 60 pulses of TMS at 120% rMT [39]) during two motor tasks (rest & hand-movement), such that participants received four distinct rounds of TMS. The order of cortical target stimulation and motor task performance was randomized on a per participant basis.

#### EEG

The 64-channel Easy EEG Cap (BrainProducts GmbH, Germany) with flat, freely rotatable, sintered Ag–AgCl electrodes specifically designed for TMS-EEG was placed and data time-locked to TMS pulses were recorded using two 32-channel TMS-compatible BrainAmp DC amplifiers (sampling rate: 5 kHz; measurement range: ± 16.384 mV; cutoff frequencies of the analog high-pass and low-pass filters: 0 and 1 kHz). Electrode impedances were below 10 kΩ. An electrode on the nasion was used as the reference. DC correction was manually triggered at the end of the stimulations at each site to prevent amplifier saturation due to DC drift.

### Data analyses

#### EEG preprocessing

EEG data analyses were performed in MATLAB (R2014b, The Mathworks Inc., MA) using custom scripts built upon the EEGLAB [40] and ARTIST [41] toolboxes. TMS-EEG data were analyzed offline with a fully-automated artifact rejection algorithm [41]. We chose to conduct all analyses for all participants at sensor level as reliable source localization is prone to error in the context of large lesions in cortical anatomy due to stroke [42]. We restricted analysis to EEG data from the bilateral primary motor cortices, which were each defined by 3 standard electrodes [Right = 4,10,22 (X:-0.195, 0.100, -0.113, Y:-0.338, -0.712, -0.926, Z:0.920, 0.694, 0.358); Left = 6,16,28 (X: -0.195, 0.110, -0.113, Y: 0.338, 0.712, 0.926, Z: 0.920, 0.693, 0.358)].

Preprocessing of the data consisted of the following steps: 1) Discard the 10 ms segments following TMS pulses to remove large stimulation-induced electric artifact and interpolate this segment with the spherical spline method [43]; 2) Downsample data to 1 kHz (lowpass filter the signal and them decimate according to the downsampled rate); 3) Remove large decay artifacts automatically using independent component analysis (ICA) based on thresholding; 4) Identify 60 Hz AC line noise artifact via the Thompson F-statistic and remove with the multi-taper regression technique. 5) Remove non-physiological slow drifts using a 0.01 Hz high-pass filter; 6) Re-reference spectrally-filtered EEG data to the common average and epoch with respect to the TMS pulse (-500 ~ 1000 ms); 7) Subtract baseline data (100 ms-300 ms pre-TMS pulse) from the entire epoch; 8) Reject bad trials by thresholding the magnitude of each trial. Reject bad channels based on thresholding the spatial correlations among channels and interpolate channels from the EEG of adjacent electrodes; 8) Remove remaining artifacts (scalp muscle, ocular, and ECG) automatically with ICA using a pattern classifier trained on expert-labeled ICs from another independent TMS/EEG data set [41].

#### EEG connectivity measurements

Beta-band (15 Hz-30 Hz spectral band) [6] connectivity was calculated on 500 ms of cleaned post-TMS-pulse EEG data. We quantified each hemisphere’s response to TMS by calculating the average of the pair-wise wPLI [30] estimates between three electrodes overlying each lateral primary motor cortex (Fig. 1) using cross-spectral density within Fieldtrip’s connectivity toolbox implementation (http://www.fieldtriptoolbox.org/). In light of our a priori hypothesis, we limited our analysis to the electrodes overlying each hemisphere’s motor cortex to improve the power of our models.

We quantified the impact of hand movement on connectivity by calculating the log transformed percent difference (ΔC) in wPLI between the two motor tasks ([wPLI Movement]/[wPLI Rest]). *The ****ΔC**** measures how much the influence of the stimulated region over the measured region changes with hand movement*.

#### Definition of experimental conditions

As we hypothesized that unilateral hand movement would increase connectivity in the **“task” hemisphere** (the hemisphere contralateral to the moving hand) more than the **“passive” hemisphere** (the hemisphere ipsilateral to the moving hand), we defined four Experimental Conditions: (1) TMS to the task motor cortex, recording over the task hemisphere (TMS-Task/EEG-Task); (2) TMS to the task motor cortex, recording over the passive hemisphere (TMS-Task/EEG-Passive); (3) TMS to the passive motor cortex, recording over the task hemisphere (TMS-Passive/EEG-Task); and (4) TMS to the passive cortex, recording over the passive hemisphere (TMS-Passive/EEG-Passive).

### Statistical analyses

#### Task-dependent changes in connectivity (ΔC)

We used a linear mixed-effects model to determine if ΔC differed between groups or across experimental conditions. Fixed factors included Group (control, stroke), Experimental Condition (TMS-Task/ EEG-Task, TMS-Task/ EEG-Passive, TMS-Passive/ EEG-Task, TMS-Passive/ EEG-Passive), and Group x Experimental Condition interaction. A random intercept of “participant” was used to account for repeated measures within participants. Degrees of freedom were adjusted via the Kenward-Roger approximation for small samples [44]. Statistically significant omnibus tests of the fixed effects were further examined via pairwise comparisons, controlling for the False Discovery Rate [45]. There was missing data for 6% of the sample, as not every subject tolerated every condition. The linear mixed-effects model uses full information maximum likelihood (FIML) to accommodate missing data, assuming it is missing at random [46]. This approach is considered a “gold standard” method to handle missing data [46]. In the post-hoc analysis, we analyzed the control and stroke data separately to determine if ΔC differed within each group between the four Experimental Conditions.

#### Clinical correlations

We examined whether change in connectivity (ΔC) was associated with Fugl-Meyer scores. This was first done via bivariate Spearman correlations. We then used an ordinary least squares regression, where upper extremity Fugl-Meyer score served as the dependent variable and Experimental Condition and lesion size served as the independent variables.

## Results

### Feasibility

TMS-EEG experiments are time-intensive, lasting 2–4 h in duration. Two patients with stroke tired before the end of the session and missed stimulation of one hemisphere (one missed ipsilesional and the other contralesional stimulation). One control also tired before undergoing stimulation of the dominant hemisphere. Otherwise, the procedure was well tolerated without concerns of side effects.

### State-dependent connectivity

The change in connectivity elicited by hand movement (ΔC) significantly differs between Experimental Conditions (F(3,90) = 3.15, *p* = 0.03), but not between stroke patients and controls (F(1,90) = 1.53, *p* = 0.22); there is no significant interaction between group and Experimental Condition (F(3,90) = 0.53, *p* = 0.66).

**Fig. 2:**
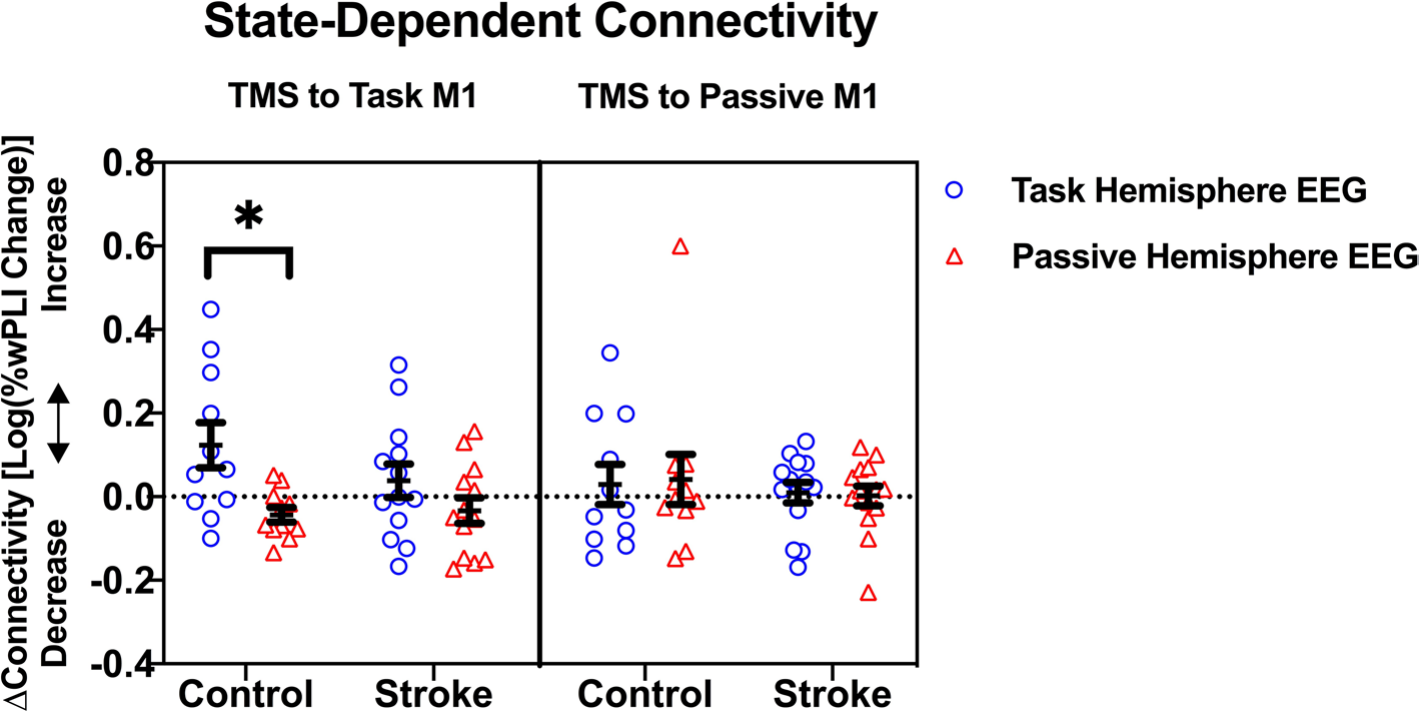
TMS-Induced Connectivity is Task Dependent in the Healthy Brain but not in Stroke. M1 = Primary Motor Cortex. *p = .03. *The ****ΔC**** measures how the influence of the stimulated motor cortex over the recorded hemisphere changes with hand movement*. Task Motor Cortex stimulation is shown in the left box. With hand movement in controls, the influence of the task motor cortex differs between the hemispheres: increased connectivity is seen within the task hemisphere (blue circles) while decreased connectivity is seen in the passive hemisphere (red triangles). With paretic hand movement in stroke patients, a similar but non-significant pattern emerges. Passive Motor Cortex stimulation is shown in the right box. Hand-movement induced connectivity differences between the hemispheres are not seen with TMS to the passive motor cortex in either group

While we did not detect a significant interaction effect between group and experimental condition, we further interrogated group differences based on our a priori hypothesis regarding stroke-specific alterations in state-dependent connectivity. Analysis of each group separately reveals that ΔC differs between Experimental Conditions within the control group, but not within the stroke group (Fig. 2). For controls, task hemisphere connectivity increases by 33% while passive hemisphere connectivity decreases by 9% (t (90) = 2.93, *p* = 0.03) when the task motor cortex is stimulated. In contrast, after passive motor cortex stimulation, ΔC does not differ between the two hemispheres (ΔC_task_ =  + 7%, ΔC_passive_ =  + 10%, t(90) = 0.21, *p* = 0.73). In stroke patients, task hemisphere connectivity also increases (ΔC_task_ =  + 9%) and passive hemisphere connectivity decreases (ΔC_passive_ = -8%), after task motor cortex stimulation, but this difference is not significant (t(90) = 1.36, *p* = 0.73). Passive motor cortex stimulation does not evoke different connectivity between the two hemispheres (ΔC_task_ =  + 7%, ΔC_passive_ =  + 10%, t(90) = 0.15, *p* = 0.92).

### Clinical correlations

**Fig. 3:**
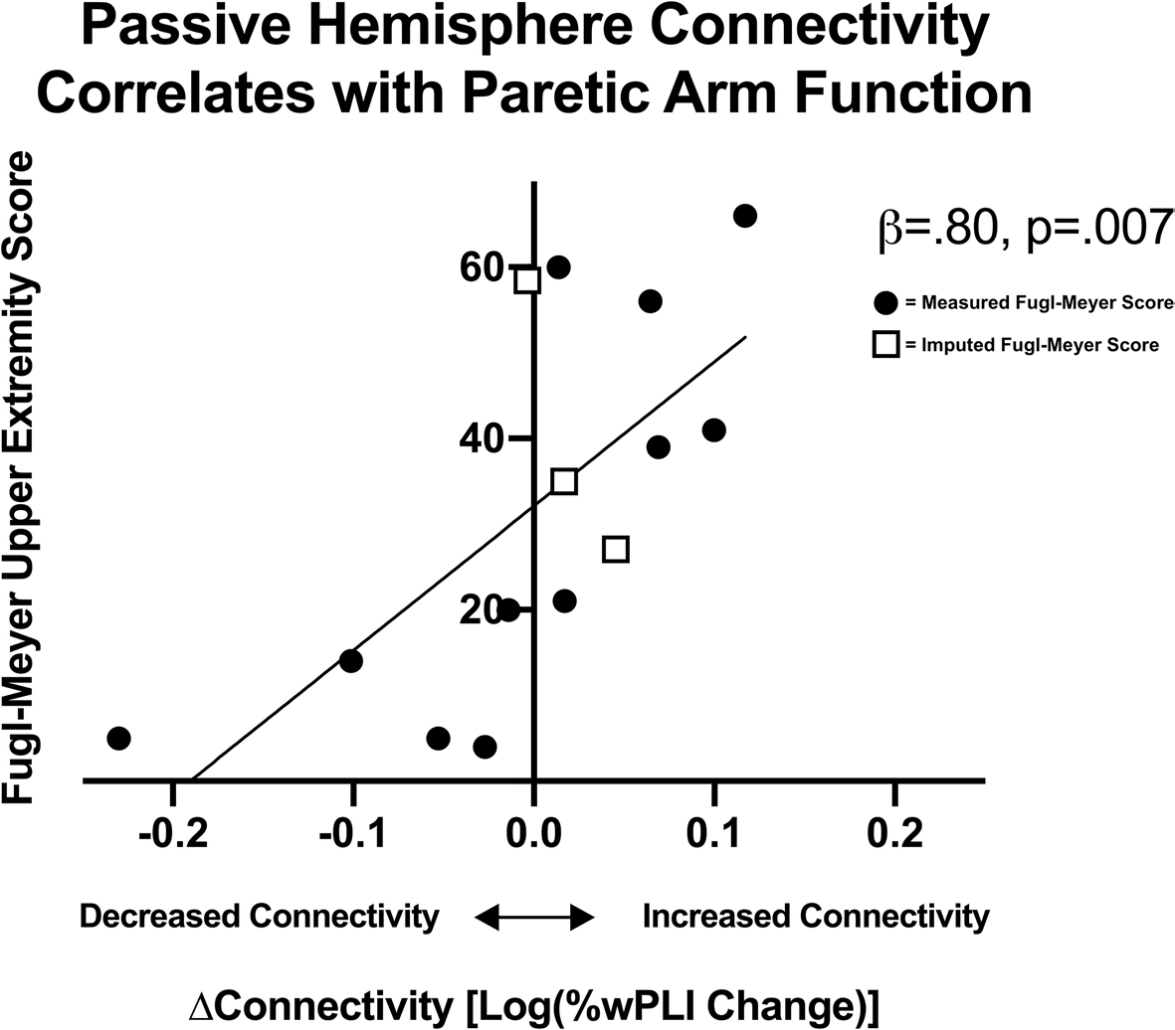
Passive Hemisphere Connectivity Correlates with Paretic Arm Function. This graph represents the change in connectivity in the passive hemisphere induced by paretic hand movement measured after passive motor cortex stimulation. Stroke patients with better paretic arm function (higher upper extremity Fugl-Meyer scores) showed an increase in connectivity within the *passive* hemisphere during paretic hand movement. Subjects who underwent a Fugl-Meyer assessment on the day of TMS are represented by closed circles and those for whom the score was imputed are represented by open squares

In stroke patients, ΔC of the passive hemisphere after passive motor cortex stimulation (TMS-Passive/EEG-Passive Condition) correlates with clinical outcomes (Fig. 3). Bivariate correlations show a strong relationship between ΔC_TMS-Passive/EEG-Passive_ and Fugl-Meyer score (Rho = 0.73, *p* = 0.005), which holds following 5,000 bootstrapped replications of the data (95% bootstrapped confidence intervals: 0.24 to 0.94). Patients with decreased passive hemisphere connectivity (ΔC_passive_ < 0) have more severe motor impairment than those with increased passive hemisphere connectivity (ΔC_passive_ > 0).There are no significant associations between Fugl-Meyer score and ΔC of the other Experimental Conditions (all Rho ≤ 0.14, *p* ≥ 0.648). The association between ΔC_TMS-Passive/EEG-Passive_ remains statistically significant even when controlling for the other three Experimental Conditions and lesion volume ($\beta$ = 0.80, *p* = 0.007). Additionally, this association remains both strong and significant if the three participants with imputed Fugl-Meyer data are excluded from the analysis ($\beta$ = 0.80, *p* = 0.027).

## Discussion

This study uses TMS-EEG to measure connectivity between the bilateral primary motor cortices in the healthy motor system and the motor system affected by stroke. We find that TMS-EEG detects connectivity changes elicited by hand movement *selectively when the task motor cortex is stimulated*: with this stimulation, connectivity within the active hemisphere increases while connectivity within the passive hemisphere decreases. Since TMS assesses the downstream influence of the stimulated cortex, this finding suggests that in controls, the task motor cortex influences brain activity bilaterally, whereas the passive hemisphere does not. In the stroke group, these task-dependent connectivity differences are attenuated. Interestingly, stroke patients with the best clinical function show a reverse pattern of connectivity. In those with the best arm function, passive motor cortex stimulation elicits a large increase in connectivity within that passive, healthy hemisphere.

Our results support our a priori hypothesis that the downstream influence of the task motor cortex changes with hand movement in healthy controls. This hypothesis was based on a large body of fMRI connectivity studies showing enhanced coupling within the task hemisphere and decreased coupling between the task and passive hemispheres during unilateral hand movement (for review see [10, 14]). Prior fMRI studies depend on effective connectivity methods that can only model causal interaction between brain regions. TMS-EEG studies add to this literature by directly testing the directionality of interactions between motor cortices. A recent study [47] of the temporal, spatial and oscillatory characteristics of TMS-EEG in controls showed that TMS elicits greater EEG activity over the stimulated hemisphere vs. the contralateral side, which they interpret as consistent with inhibition of the contralateral hemisphere by the stimulated one. Our study extends upon this prior work by examining how motor tasks alter interhemispheric interactions measured by TMS-EEG. In controls, task-dependent connectivity changes are seen only when the task hemisphere is stimulated. This suggests that the task hemisphere exerts influence bilaterally during movement, whereas the passive hemisphere becomes functionally isolated, consistent with prior work showing that output from the passive hemisphere is inhibited during movement [48].

We find no significant connectivity differences between the stroke and control groups or within the stroke group. Task-dependent connectivity changes are not elicited by passive hemisphere stimulation, contradicting our hypothesis that the healthy passive hemisphere inhibits the lesioned task hemisphere during movement. If anything, connectivity in the stroke group resembles the pattern seen in controls, with more striking changes elicited by lesioned task hemisphere stimulation, though this is not statistically significant. Why do our results differ from prior work showing that the healthy, passive hemisphere inhibits the injured one during paretic hand movement? One possibility is that our group differs from previously studied stroke patients. The majority of our subjects had lesions affecting both the cortex and subcortical regions where as many prior studies focused on patients with only subcortical injury [5]. Other authors have found that hemispheric interactions differ in patients with mixed cortical-subcortical vs. only subcortical injury [49, 50], and that response to neuromodulatory therapy also vary based on lesion location [8]. A second interpretation is that the inhibitory influences from the healthy to the lesioned hemisphere seen with fMRI [8] are indirect – occurring via input from the premotor and supplementary motor area – and hence are not detected in the milliseconds following the TMS pulse. Our methodology, which examines connectivity during a period of hand movement rather than indexed against each individual hand movement, is more akin to that used in fMRI studies than prior TMS studies [12]. A third consideration is that the passive hemisphere might have been stimulated at relatively high intensities, because we based stimulation intensity for both hemispheres on the motor threshold of the affected hemisphere, which may have been elevated due to the stroke. It is possible that suprathreshold stimulation of the passive hemisphere obscured subtle movement-induced changes in connectivity. Future TMS-EEG studies could stimulate at various times prior to and following hand movement to more precisely map information flow within the motor network.

Only a handful of studies have used TMS-EEG to study stroke. Two studies demonstrated that the magnitude, latency, and distribution of early TMS-EEG-evoked potentials (TEPs) [51, 52] elicited during motor tasks differs in chronic stroke patients compared with controls and that TEPs correlate with upper extremity function in the stroke population. Other authors have identified that TMS-EEG data can predict motor recovery over time [53, 54]. Extending beyond the TEP methodology, Pellicciari et al. [53] found that during a brief window of early recovery, increased alpha oscillations are induced by stimulation of ipsilesional vs. contralesional motor cortex. As the power of these oscillations relates to clinical recovery, the authors conclude that hemispheric-specific oscillations are a biomarker of cortical reorganization. This work however was limited to the resting state. A single study investigates the impact of paretic hand movement on connectivity metrics [6]. Here, subjects performed a transcallosal inhibition task while TMS was applied to the hemisphere ipsilateral to tonic hand contraction. The investigators found an increase in connectivity *between* the two motor cortices with stimulation of the lesioned hemisphere in stroke patients when compared to stimulation of the non-dominant (but not the dominant) hemisphere of controls. Therefore, it is difficult to conclude that these findings are due to stroke rather than secondary to hemispheric dominance. In contrast to our methodology, this study measured connectivity between (rather than within) the hemispheres and focused on absolute connectivity (rather than change in connectivity induced by movement).

In concordance with the aforementioned TMS-EEG work, we also find that TMS-EEG connectivity measures correlate with clinical function after stroke. After controlling for stroke volume, subjects with the largest increase in healthy passive hemisphere connectivity induced by movement have the best upper extremity function. This correlation is selectively associated with passive hemisphere stimulation and not seen with task hemisphere stimulation. One possible interpretation of this finding is that well-recovered patients migrate cortical control of the paretic hand to the healthy passive hemisphere. This is supported by the fact that in controls, stimulation of the active motor cortex controlling the hand movement leads to a selective increase in connectivity. This interpretation is controversial as it contradicts much of the fMRI literature, which has found that patients with increased BOLD signal in the healthy hemisphere during paretic hand movement have worse functional outcomes (for review see [55]). However, fMRI studies focusing on severely affected stroke populations have shown correlations between contralesional activation and better outcomes [55–59], suggesting that the healthy hemisphere supports movement in at least some patients. Another consideration is that our connectivity measure (wPLI) quantifies changes in neuronal synchronization, but does not distinguish between excitation or inhibition of the neural population. Therefore, an alternative interpretation of our finding is that patients with better clinical outcomes develop stronger inhibition of the contralesional hemisphere during movement. Further studies can investigate the neural underpinnings of these connectivity changes by incorporating paired-pulse TMS methods. Even without this physiologic insight, however, TMS-EEG markers of connectivity may provide useful biomarkers of brain state after stroke against which therapeutic rTMS protocols could be assessed.

## Limitations

Our findings complement the existing literature, but several methodological factors limit interpretation. First, our small sample size limits our ability to detect higher order interactions. While the heterogeneity of our stroke group – with both cortical and subcortical strokes – improves generalizability of our results, the variability limits our power to detect differences. Larger studies could assess how size or location of stroke impact connectivity.

There are also several limitations to our experimental paradigm. First, we only asked subjects to move one hand. Control subjects moved their dominant (right) hand while stroke subjects (who were all initially right-hand dominant) moved their paretic hand. We collapsed left and right hemispheric stroke patients into one group and defined connectivity relative to the task (ipsilesional) vs. passive (contralesional) hemispheres. This approach, while maximizing our power, limits our ability to speak to hemispheric-specific differences in connectivity; this is particularly relevant given the Borich et al. [6] results. A replication study in which subjects move both hands could assess if connectivity differences are specific to the dominant side or are generalizable bilaterally and if hemispheric dominance impacts connectivity after stroke. Second, the motor task was not carried out in precisely the same way for each participant. Subjects were not asked to perform the task at a standardized frequency as that would have required differential effort depending on an individual’s motor ability, which itself would have introduced a confound. Given this, we did not account for the effect that frequency of the task may have on motor cortex connectivity. Additionally, 57% of stroke patients could not move their paretic hand and hence visualized the movement. While it has been shown that motor imagery elicits comparable primary motor cortex activation as actual movement [38], it is possible that this difference in the task affected connectivity. To assess this, we stratified stroke subjects on Fugl-Meyer Score but found no significant differences in connectivity between strata (analysis not shown).

Another potential concern is that we do not account for the severity of motor deficits in our primary analysis. Interhemispheric dynamics change depending on the severity of the motor deficit [5, 60, 61]. Thus, several researchers have proposed the bimodal-balance recovery hypothesis [62, 63], which states that structural reserve of the ipsilesional hemisphere determines whether the ipsilesional or contralesional hemisphere supports motor function. We found no differences in connectivity when stratifying participants by Fugl-Meyer Score, but this secondary analysis was underpowered. Though we find that connectivity varies with clinical function in a linear fashion, our analysis may miss more complex interactions. Larger future studies should consider such interactions in determining appropriate power. It is worth noting that while the current study focused on chronic stroke, further investigation of state-dependent cortical connectivity in acute and sub-acute phases of stroke recovery as it relates to healthy controls would provide valuable insight into the temporal trajectory of cortical dynamics following stroke, and inform early-intervention efforts. Finally, certain medications, including medications to treat seizures, spasticity and pain, can alter cortical excitability. While heterogeneity in prescription medication makes controlling for these effects difficult, we feel keeping our primary outcome a within-subject comparison of “active” versus “passive” motor condition, and obtaining an individualized threshold for each subject, helps to attenuate the influence various medication pose to our results.

There are several inherent limitations to TMS and EEG that impact interpretation our results. Though it provides high temporal resolution, EEG lacks spatial resolution and is susceptible to volume conduction as signal is measured at the scalp rather than the surface of the brain [64]. One method to address these pitfalls is to do source localization of the data. However, we opted against this method to limit bias that would be differentially introduced when doing source analysis on intact vs. lesioned brains. Thus our interpretational specificity is limited to the motor region in general, as opposed to quantifying premotor or primary motor specific effects. Fortunately, connectivity methods such as wPLI robustly account for volume conduction [30], and hence this is unlikely to significantly confound our data. Further, while a powerful causal investigation tool, TMS is limited in both its cortical reach and spatial specificity [65]. Though TMS-EEG is a robust methodology in causal imaging, interpretation is limited by the lack of understanding as to the neural origins of the TMS-evoked EEG metrics [66].

Several studies have established that TEPs are highly reliable and reproducible [39, 67, 68], and a more recent study [47] reported that temporal, spatial and oscillatory TMS-EEG measurements also have high reproducibility across individuals and high test–retest reliability within individuals. However, we cannot assume this to be the case in stroke patients. For example, while motor evoked potentials (MEPs) are also highly reproducible in controls, a number of studies [69] have found that they are not always reproducible after stroke. Theoretically, however, TMS-EEG signals which depend primarily on integrity of the cortex may be more reliable measures of the neurophysiology of the surviving cortex after stroke than MEPs which rely on the integrity of the entire corticospinal tract. Future studies will be needed to delineate this.

Lastly, it is worth noting that while we took methodological steps to minimize peripherally-evoked potentials (including white-noise masking and foam to dampen vibrations) [21], a number of recent studies have demonstrated similarities between TEPs following TMS and control conditions despite these masking procedures [66, 70]. Therefore, we cannot entirely rule-out the possibility that the TMS-evoked oscillations we see are truly transcranial rather than secondary to multisensory artifacts [66]. However, we believe these effects are minimized by the within-subject design of our tested hypothesis such that all analyses were contrasts between rest and movement states. It is also reassuring that the increase in connectivity we see in the active hemisphere occurs specifically with stimulation of that hemisphere, as “clearly lateralized” TEPs “confined to the stimulation site” [66] are less likely to be artifactual. Furthermore, wPLI is robust not only against volume conduction of intracranial signal but also similar zero phase-lag signal that could be created by muscle, ocular, sensory, and auditory artifacts [71].

## Conclusion

The current results provide a promising step in characterizing the causal circuit dynamics within the motor system, their reorganization following stroke, and how this relates to motor recovery. Importantly, we demonstrate that TMS-EEG metrics capture task-dependent changes in brain dynamics and that, in controls, these changes are seen specifically when stimulating the hemisphere engaged in the motor task. Furthermore, we find a robust correlation between a TMS-EEG connectivity measure and clinical function of stroke patients, suggesting that TMS-EEG connectivity metrics may provide good biomarkers for assessing brain dynamics after stroke. While future work can focus on clarifying the physiological underpinnings of these connectivity changes, it arguably will be more important to develop these metrics as biomarkers that can be incorporated into rTMS therapeutic trials. The availability of such intermediary biomarkers could ultimately allow for rapid and personalized selection of rTMS parameters, including preferred site and experimental task for stimulation.

## Data Availability

The datasets used and/or analysed during the current study are available from the corresponding author on reasonable request.
